# Immunosuppressive therapy in inflammatory ocular surface disease post Steven Johnson syndrome

**DOI:** 10.4103/0301-4738.73701

**Published:** 2011

**Authors:** C Balkrishnan, Vishnu Sharma, Anupama Vyas

**Affiliations:** Department of Rheumatology, P D Hinduja National Hospital and MRC, Mumbai, India

Dear Editor,

Stevens-Johnson syndrome (SJS) is an acute inflammatory disease which often affects the skin and mucosal membranes including that of eyes.[1] On recovery, up to 50% of the patients with SJS develop chronic ocular surface problems which may need immunosuppressive therapy.[2] We report our experience with seven patients of SJS with ocular complications who were treated with systemic immunosuppressive therapy. These patients were referred to us by the treating ophthalmologist because local therapy had not helped and they felt that there was chronic inflammation which could respond to systemic immunosuppressive therapy. We are reporting these cases due to the rarity of the condition and lack of data in the literature.

The details of the seven patients are summarized in the Table 1. SJS was secondary to a viral illness in three patients, in two patients it was due to an allergic reaction to sulpha drugs and it was secondary to phenytoin and carbamazepine therapy in one each. Persistent dryness and ocular surface inflammation in spite of local therapy was the presenting symptom in all patients. Other ophthalmic findings were not given to us by the treating ophthalmologist. Laboratory parameters (complete hemogram, erythrocyte sedimentation rate (ESR), liver function tests, renal function tests, urine routine) were normal except for unexplained increase in ESR in two patients.

**Table 1:** Immunosuppressive therapy in post Steven Johnsons Syndrome inflammatory ocular surface disease

Age (Yrs)/Sex	Year of onset	Date of 1^st^ visit	IS therapy	Duration of therapy	Outcome**
21/F	2004	23/3/06	Pred + MTX	6 months	No improvement
42/F	Dec 2001	29/4/04	MTX	10 months	No improvement
24/M	May 2004	11/9/04	MTX	9 months	Good improvement
31/F	1983	8/4/08	Pred + AZA	1 year	Partial improvement
45/F	April 2004	12/10/04	Oral CYC followed by MTX	2.5 years	Partial improvement
18/M	1992	22/10/02	MTX	6 months	Partial improvement
30/F	Sept 2002	18/2/03	Pred*+ MTX	1 year	Partial improvement

MTX: methotrexate, Dose 7.5 mg to 15 mg, AZA: azathioprine, Dose 2-3 mg/kg body weight, CYC: cyclosporine, Dose 2.5 to 4 mg/kg per day, M: male, F: female,

* Prednisolone was given orally at 0.5 mg/kg/day for four weeks followed by taper to maintenance of 5-7.5 mg/d,

** Outcome: Based on Ophthalmologist’s opinion and patient subjective improvement, No improvement - < 25%, Partial improvement – 26% to 50%, Good improvement – >50%

Oral steroids were the cornerstone of treatment. Steroid sparers were added if steroid alone did not control the inflammation. The choice of steroid sparer was empirical as there are no established guidelines. Methotrexate has been established to treat a variety of inflammatory eye diseases hence it was used in most of the patients. Patient 5 was on topical cyclosporine, since there was no response he was switched to oral cyclosporine which was later stopped due to intolerance. Duration of the treatment was according to patient response and tolerance to drugs. At a mean follow-up of 10.42 months (range 6-30 months) there was no treatment-related complication.

Chronic ocular surface complications are known to occur after SJS. These include symblepharon, entropion, trichiasis, dry eyes, persistent conjunctival inflammation, conjunctival injection and corneal opacification.[2,3] A recent article classifies these complications as Stevens-Johnson ocular surface failure (SJS-OSF), Stevens-Johnson recurrent inflammation (SJS-RI), Stevens-Johnson ocular membrane pemphigoid (SJS-MMP) and Stevens-Johnson scleritis (SJS-scleritis).[3]

The exact pathogenesis of these lesions is unknown. Destruction of the limbus during the acute stage can lead to stem cell deficiency. Although the cornea looks normal after the acute stage abates, the peripheral limbal cells slowly fail over a period of time, sometimes leading to complete surface failure in a few years. Further, some patients also develop prolonged limbal inflammation which eventually leads to limbal cell failure. Kawasaki *et al* studied the conjunctiva of five patients with chronic ocular problems post SJS. They found that the substantia propria of the conjuctivalised cornea was infiltrated with CD4-positive T-cells, CD8-positive T-cells and macrophages. Further, there was predominant interferon gamma production at the tissue level suggesting that the Th1 axis of the immune system was active. In these patients very little conjunctival inflammation was clinically evident.[4]

Conjunctival inflammation in these patients may be due to trichiasis or severe dry eye. These have to be treated effectively by local measures. If in spite of this the inflammation persists and local measures fail, then there is a role for systemic medications. Systemic steroids and various other steroid-sparing agents including cyclosporine, azathioprine, cyclophosphamide, methotrexate have been used with varying success.[3]

In our patients, the time lag between the SJS and the late ocular complication varied from a few months to many years. All these patients had not responded to local therapy. The response to oral steroids and other immunosuppressive therapy was not predictable. Except for two patients who showed no response, other patients followed with us at least for six months after stopping therapy. The response was maintained even after stopping immunosuppressive therapy. There are limitations in this study. This is a retrospective observational study. Due to this objective improvement criteria were not used.

In conclusion post-SJS dry eyes is an important condition causing significant morbidity. Its pathogenesis is still not well understood. If local causes are ruled out and local treatments fail, there could be a role for systemic immunosuppressive therapy. There is a need for controlled trials to find out which immunosuppressive therapy best benefits these patients.
